# Description of a new clinical syndrome: thoracic constriction without evidence of the typical funnel-shaped depression—the "invisible" pectus excavatum

**DOI:** 10.1038/s41598-023-38739-w

**Published:** 2023-07-25

**Authors:** Anna Hohneck, Uzair Ansari, Michèle Natale, Karsten Wittig, Daniel Overhoff, Philipp Riffel, Michael Boettcher, Ibrahim Akin, Daniel Duerschmied, Theano Papavassiliu

**Affiliations:** 1 Department of Cardiology, Angiology, Haemostaseology and Medical Intensive Care, University Medical Centre Mannheim, Medical Faculty Mannheim, Heidelberg University, Theodor-Kutzer-Ufer 1-3, 68167 Mannheim, Germany; 2 European Center for AngioScience (ECAS) and German Center for Cardiovascular Research (DZHK) partner site Heidelberg/Mannheim, Mannheim, Germany; 3 Department of Clinical Radiology and Nuclear Medicine, University Medical Centre Mannheim, Medical Faculty Mannheim, Heidelberg University, Mannheim, Germany; 4 Department of Pediatric Surgery, University Medical Centre Mannheim, Medical Faculty Mannheim, Heidelberg University, Mannheim, Germany

**Keywords:** Cardiology, Medical research

## Abstract

Pectus excavatum (PE) is a congenital malformation with a funnel-shaped depression of the sternum that can lead to cardiac symptoms. However, there are patients with thoracic constriction (defined as elevated Haller-Index > 3.25 determined by cardiac magnetic resonance imaging (CMR)) without visible evidence of PE, leading to similar complaints. Between January 2004 till June 2020, patients who underwent CMR for further evaluation of the heart, due to cardiac symptoms were enrolled and compared to controls. Biventricular global strain analysis was assessed using feature tracking (CMR-FT). ECG and/or Holter recordings were performed to detect rhythm events. Cardiac symptoms were evaluated in detail using a questionnaire. Finally, 88 patients (male 35, female 53) with elevated Haller-Index (3.9 ± 0.8) were included and compared to CMR data from 25 individuals with confirmed PE and 25 healthy controls (HC). Mean age at time of CMR was 35 ± 16 years. The most common symptoms at presentation were palpitations (41%), followed by dyspnea (24%) and atypical chest pain (14%). Three patients (3%) had atrial fibrillation or atrial flutter. Concomitant phenomena were pericardial effusion in 39% and mitral valve prolapse (MVP) in 27% of the study cohort. While there were no differences in left ventricular function or volumes, right ventricular function (RVEF) was significantly lower in patients with internal PE compared to HC (RVEF (%) 50 ± 5 vs 59 ± 4, p < 0.01). Strain analysis revealed only discrete changes in RV strain, implying a purely mechanical problem in the absence of structural changes. RV dimensions were negatively correlated with the size of thoracic indices (r = 0.41), reflecting the extent of thoracic constriction. MVP was more prevalent in patients with greater thoracic indices (r = 0.24). The described cohort, referred to as internal PE because of the absence of external changes, showed similar CMR morphologic findings as patients with real PE (especially altered dimensions of the right heart and a lower RVEF). In addition, there was a high incidence of rhythm disturbances, such as extrasystoles or arrhythmias. In one-third of the study cohort additional abnormalities such as pericardial effusion or MVP were present, with MVP being found more frequently in patients with larger thoracic indices, suggesting a possible common pathogenesis.

**Trial registration:** ISRCTN registry, ISRCTN15355937, retrospectively registered 03.06.2022, https://www.isrctn.com/ISRCTN15355937?q=15355937&filters=&sort=&offset=1&totalResults=1&page=1&pageSize=10.

## Introduction

Pectus excavatum (PE) is a congenital deformity that leads to a funnel-shaped depression of the anterior chest wall^1^. This pathognomonic feature gives the disease its common name, “funnel chest”. For a long time, it was assumed that PE was an inconsequential condition with symptoms rather of cosmetic character. However, the deformity can lead to reduced physical performance, especially during exercise, mainly caused by compression of the right ventricle (RV)^2, 3^. In several studies, reduced lung volumes associated with lower airway obstruction could also be observed^4–6^.

The quantification of a PE is performed using thoracic imaging to determine the Haller-Index^7^. The Haller-Index is defined as the ratio of the transverse thoracic diameter divided by the frontodorsal diameter of the chest. Normal values are < 3.25, with higher values indicating greater severity of PE^8^. While the Haller index depends on the width and does not assess the depth of the defect, other indices have been developed, including the Correction-Index^9^ and the Depression-Index^10^, to better reflect the dimensions.

Echocardiographic assessment, especially of the RV, can be difficult in patients with PE, due to the anatomical conditions^11^. Cardiovascular magnetic resonance imaging (CMR) on the other hand, can provide further insights. CMR is a relatively robust and examiner-independent method, which is able to assess both morphology and function of PE in detail^12^. Both ride-sided heart anomalies, such as tricuspid valve prolapse or mildly reduced RV function and pericardial effusion are common findings revealed by imaging modalities in patients with PE^13^. In addition, CMR based myocardial strain analysis (feature tracking, FT) has emerged as a promising technique detecting subtle functional changes and structural abnormalities, before a deterioration of the cardiac function becomes apparent.

While the diagnosis of PE typically can be made at a glance, there are patients who show only discrete indications to no visible external changes, and an unobtrusive echocardiography, but present with non-specific cardiac symptoms. In CMR, however, there is evidence of a “PE”, with an increased Haller index, but without the typical funnel-shaped sternal depression. The current paper presents a case series of patients with cardiac symptoms who have received a CMR for work-up, showing thoracic constriction in terms of an "invisible" PE.

## Results

### Baseline characteristics

#### Study population

**Figure 1:**
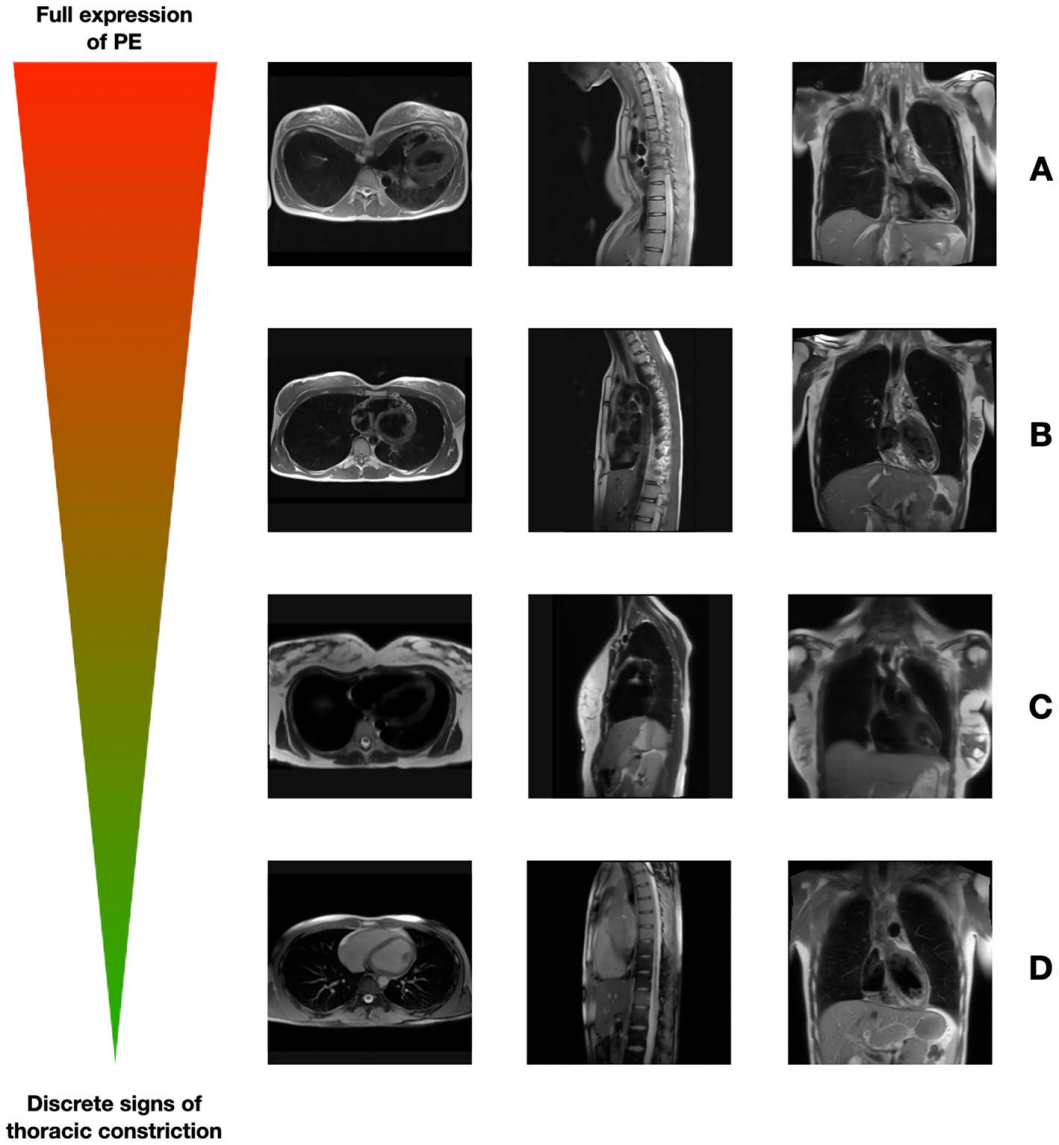
Comparative display of patients with different expressions of PE. The figure shows different CMR scans with transverse, sagittal and coronal views of the sternal region of (**A**) a patient with a real PE and complete left shift of the heart compared to (**B**) a patient with a severe form of internal PE, without evidence of the typical funnel-shaped depression. Figures (**C**) and (**D**) also show relative thoracic constriction, with varying degrees of severity and thus varying burden of symptoms.

88 patients are reported in this analysis, 53 of whom are female (60%). Mean age at time of CMR was 35 ± 16 years. In 78 individuals CMR was performed due to non-specific cardiac symptoms (palpitations (n = 36, 41%), dyspnea (n = 21, 24%), atypical chest pain (n = 12, 14%) and syncope (n = 9, 10%)) with unremarkable findings in echocardiography, while 8 patients (9%) had echocardiographical indications of a cardiomyopathy (CMP). Two patients without proven CMP or channelopathy, but mitral valve prolapse (MVP), had experienced ventricular fibrillation (VF, 2%). All patients showed no visible or only discrete signs of a PE (compare Fig. 1: Different expressions of PE).

85 patients (97%) had a documented sinus rhythm in ECG, while 2 patients had atrial fibrillation (2%) and another atrial flutter (1%), with a mean heart rate of the study population of 71 ± 21 bpm. Supraventricular extrasystoles (SVES) were present in 13 (15%) and ventricular extrasystoles (VES) in 18 (21%) patients, while a complete left bundle branch block (n = 3) or right bundle branch block (n = 1) was found in four (5%) and an incomplete right bundle branch block in six (7%) individuals.

#### Morphological findings

The most common morphological finding was pericardial effusion, which was present in 39% (n = 34), followed by MVP (n = 24, 27%) and other cardiac vitia (n = 10, 11%). Scoliosis was present in three patients (3%).

Complete baseline characteristics are shown in Table 1.

**Table 1:** Baseline characteristics of patients with internal PE (n = 88).

	Internal PE (n = 88)
Age at time of presentation (years)	35.0 ± 15.8
Age min, max (years)	14, 77
Sex, male	35 (40)
ECG	
HR (bpm)	71 ± 21
SR	85 (97)
Atrial fibrillation	2 (2)
Atrial flutter	1 (1)
SVES	13 (15)
VES	18 (21)
Complete left bundle branch block	3 (3)
Complete right bundle branch block	1 (1)
Incomplete right bundle branch block	6 (7)
Symptoms at presentation	
Palpitations	36 (41)
Dyspnea	21 (24)
Atypical chest pain	12 (14)
Syncope	9 (10)
Echocardiographic findings of CMP	8 (9)
Ventricular fibrillation	2 (2)
Morphological findings	
Pericardial effusion	34 (39)
Mitral valve prolapse	24 (27)
Cardiac vitium	10 (11)
Scoliosis	3 (3)

Data are presented as mean ± standard deviation or numbers (frequencies).

*bpm* beats per minute, *CMP* cardiomyopathy, *ECG* electrocardiogram, *HR* heart rate, *PE* pectus excavatum, *SR* sinus rhythm, *SVES* supraventricular extrasystole, *VES* ventricular extrasystole.

### CMR characteristics

#### PE characteristics of patients with an internal PE

The extent and severity of PE was evaluated using the thoracic indices. Patients with an internal PE showed a mean Haller-Index of 3.9 ± 0.8 and a mean Correction-Index of 14.4 ± 9.9. Depression-Index, on the other hand, was less striking, with a mean value of 0.2 ± 0.2. The mean contact zone of the heart was 81 ± 27 mm. Intracardiac angles ranged from 43° (RV apex) to 57° (LV apex) (Table 2).

**Table 2:** Comparison of CMR characteristics and clinical findings of patients with internal PE (n = 88), patients with real PE (n = 25) and healthy controls (HC, n = 25).

	Internal PE (n = 88)	Real PE (n = 25)	HC (n = 25)	p-value internal PE vs real PE	p-value internal PE vs HC	p-value real PE vs HC
Age at time of CMR (years)	35.0 ± 15.8	29.6 ± 15.0	34.7 ± 7.4	0.13	0.89	0.28
Sex, male	35 (40)	13 (52)	13 (52)	0.28	0.28	1.00
CMR						
Left heart						
LV-EF (%)	58 ± 6	60 ± 9	59 ± 5	0.21	0.14	1.00
LV-EDVi (ml/m^2^)	82 ± 18	86 ± 17	74 ± 11	0.37	**0.04**	**0.01**
LV-ESVi (ml/m^2^)	35 ± 11	34 ± 10	30 ± 6	0.60	**0.03**	0.14
LV-SVi (ml/m^2^)	47 ± 10	52 ± 11	44 ± 8	0.06	0.11	** < 0.01**
MAPSE septal (mm)	14 ± 4	14 ± 5	16 ± 3	0.40	**0.02**	0.28
LV-GRS (%)	36 ± 11	36 ± 12	37 ± 12	0.93	0.64	0.68
LV-GCS (%)	− 19 ± 4	− 19 ± 4	− 19 ± 2	0.87	0.99	0.89
LV-GLS (%)	− 13 ± 3	− 13 ± 3	− 14 ± 3	0.84	0.76	0.68
Right heart						
RV-EF (%)	50 ± 5	50 ± 5	59 ± 4	0.85	** < 0.01**	** < 0.01**
RV-EDVi (ml/m^2^)	86 ± 17	88 ± 20	69 ± 13	0.60	** < 0.01**	** < 0.01**
RV-ESVi (ml/m^2^)	44 ± 11	45 ± 13	29 ± 7	0.68	** < 0.01**	** < 0.01**
RV-SVi (ml/m^2^)	42 ± 8	44 ± 9	40 ± 8	0.58	0.15	0.13
TAPSE (mm)	22 ± 5	17 ± 4	22 ± 3	** < 0.01**	0.80	** < 0.01**
RV-GRS (%)	24 ± 17	20 ± 5	27 ± 14	0.27	0.36	**0.02**
RV-GCS (%)	− 11 ± 3	− 9 ± 6	− 12 ± 3	0.36	0.15	0.08
RV-GLS (%)	− 11 ± 10	− 10 ± 12	− 15 ± 4	0.59	**0.03**	**0.02**
Right ventricular dimensions (mm)						
RV-EDD basal	40 ± 9	31 ± 8	43 ± 6	** < 0.01**	0.23	** < 0.01**
RV-ESD basal	30 ± 7	25 ± 6	25 ± 4	** < 0.01**	** < 0.01**	0.78
RV-EDD midventricular	29 ± 7	30 ± 6	33 ± 5	0.47	** < 0.01**	**0.03**
RV-EDD apical	18 ± 5	24 ± 5	19 ± 4	** < 0.01**	0.19	** < 0.01**
RV-EDD longitudinal	90 ± 13	96 ± 10	77 ± 8	**0.03**	** < 0.01**	** < 0.01**
Right atrial dimensions (mm)						
RAD major	41 ± 10.6	36 ± 11	37 ± 5	0.053	0.13	0.54
RAD minor	40.1 ± 7.6	36 ± 8	33 ± 6	**0.02**	** < 0.01**	0.23
LGE	12 (14)	3 (12)	0 (0)	0.83	0.051	0.08
PE characteristics						
Haller-Index	3.9 ± 0.8	6.6 ± 2.7	2.6 ± 0.5	** < 0.01**	** < 0.01**	** < 0.01**
Depression-Index	0.2 ± 0.2	1.0 ± 0.3	0.1 ± 0.1	** < 0.01**	** < 0.01**	** < 0.01**
Correction-Index	14.4 ± 9.9	44.7 ± 11.5	3.5 ± 4.6	** < 0.01**	** < 0.01**	** < 0.01**
Contact zone (mm)	81 ± 27	95 ± 23	44 ± 15	**0.02**	** < 0.01**	** < 0.01**
Intracardiac angles						
Angle Apex-RV	43 ± 11	53 ± 11	37 ± 7	** < 0.01**	**0.01**	** < 0.01**
Angle insertion	50 ± 11	61 ± 10	45 ± 7	** < 0.01**	**0.03**	** < 0.01**
Angle Apex-LV	57 ± 11	66 ± 10	53 ± 6	** < 0.01**	0.08	** < 0.01**
Mitral valve						
Length of anterior mitral leaflet (mm)	26 ± 4	27 ± 5	24 ± 4	0.23	**0.02**	**0.01**
Mitral valve prolapse	24 (29)	4 (16)	0 (0)	0.25	** < 0.01**	**0.04**
Other findings						
Pericardial effusion	34 (41)	6 (24)	0 (0)	0.18	** < 0.01**	**0.01**
Cardiac arrhythmia	45 (54)	10 (40)	0 (0)	0.94	** < 0.01**	** < 0.01**

Data are presented as mean ± standard deviation or numbers (frequencies). Volumes are indexed to body surface area (i). Comparisons between three groups were performed with ANOVA and Scheffé post Hoc test. Bold values indicate statistical significance.

*CMR* cardiac magnetic resonance imaging, *EDD* enddiastolic diameter, *EDV* enddiastolic volume, *EF* ejection fraction, *ESD* endsystolic diameter, *ESV* endsystolic volume, *GCS* global circumferential strain, *GLS* global longitudinal strain, *GRS* global radial strain, *HC* healthy controls, *LGE* late gadolinium enhancement, *LV* left ventricle, *PE* pectus excavatum, *RAD* right atrial diameter, *RV* right ventricle, *SV* stroke volume.

#### Comparison of PE characteristics of patients with an internal PE (n = 88) with patients with a real PE (n = 25) and HC (n = 25)

Thoracic indices that measure the severity of PE were all significantly greater in patients with a real PE (p < 0.01 vs internal PE and HC), although patients with an internal PE also had significantly greater thoracic indices compared with HC (p < 0.01). Similar results were found for the intracardiac angles, which were largest in patients with a real PE and ranged from 53° (RV apex) to 66° (LV apex).

HC showed the smallest contact zone with mean values of 44 ± 15 (p < 0.01 vs patients with internal and real PE).

#### Functional CMR findings of patients with an internal PE

Compared to age-referenced normal values, patients with an internal PE showed normal LV function and volumes (LVEF (%) 58 ± 6). RV function, on the other hand, was slightly reduced, though within the normal range (RVEF (%) 50 ± 5) with correspondingly higher end-systolic volumes (44 ± 11 ml/m^2^). Tricuspid annular plane systolic excursion (TAPSE) was normal (22 ± 5 mm). In 2D-measurements, RV was enlarged (end-diastolic diameter (EDD) basal 40 ± 9 mm; end-systolic diameter (ESD) basal 30 ± 7 mm) with a tubular configuration, resulting in an expansion of the apical diameter (RV-EDD apical 18 ± 5 mm). The RA diameters (RAD) were also enlarged (RAD major 41 ± 11 mm; RAD minor 40 ± 8 mm). LGE was present in 12 patients (14%), which was classified as non-ischaemic in 83% of patients (10 out of 12) due to intramyocardial localization (myocarditis n = 3, MVP n = 3, dilatative CMP n = 1, hypertrophic CMP n = 1, coronary embolism n = 1, after ablation n = 1). Two patients were diagnosed with coronary artery disease.

#### Comparison of functional CMR findings of patients with an internal PE (n = 88) with patients with a real PE (n = 25) and HC (n = 25)

CMR characteristics and clinical findings of patients with an internal PE were then compared to patients with confirmed PE (13 men and 12 women, age 30 ± 15 years) and healthy controls (13 men and 12 women, age 35 ± 7 years), matched for age and sex.

While there were no differences in LVEF between patients with internal or real PE and HC, LV volumes (EDV and ESV) were elevated when compared to HC (p < 0.05). MAPSE was lower in patients with an internal PE compared to HC ((mm) 14 ± 4 vs 16 ± 3, p = 0.02). Striking differences became apparent regarding RV function and volumes. Lower RVEF (still within the normal range) could be observed in both patients with internal or real PE compared to HC (RVEF (%) internal PE 50 ± 5 vs HC 59 ± 4, p < 0.01; real PE 50 ± 5 vs HC 59 ± 4, p < 0.01), with significantly increased volumes. While TAPSE did not differ between patients with internal PE and HC, TAPSE was reduced in patients with real PE in comparison to patients with internal PE and HC (TAPSE (mm) internal PE 22 ± 5 vs real PE 17 ± 4, p < 0.01; real PE 17 ± 4 vs HC 22 ± 3, p < 0.01).

Under normal circumstances, the RV forms the shape of a triangle, with the basal diameter of the RV being the largest and decreasing towards the apex. In patients with a real PE, however, sternal retraction results in compression of the RV, giving it an elongated configuration with a midventricular retraction, and a relatively large apical diameter. While basal diameters of internal PE and HC did not differ, patients with a real PE had a significantly smaller basal RV-EDD (p < 0.01) compared to patients with internal PE and HC. Against this, midventricular RV-EDD of both patients with an internal and real PE were smaller compared to HC ((mm) internal PE 29 ± 7 vs HC 33 ± 5, p < 0.01; real PE 30 ± 6 vs HC 33 ± 5, p = 0.03). Largest apical diameters were found in patients with real PE with mean values of 24 ± 5 mm (p < 0.01 vs internal PE and HC).

The RA on the other hand was enlarged in patients with internal PE. This was particularly evident regarding RAD minor ((mm) internal PE 40 ± 8 vs real PE 36 ± 8, p = 0.02; internal PE 40 ± 8 vs HC 33 ± 6, p < 0.01).

LGE was detected in both patients with internal and real PE (internal PE n = 12 (14%) vs real PE 3 n = 3 (12%), p = 0.83), whereas there was no evidence of LGE in HC.

##### Morphological findings

Pericardial effusion or cardiac arrhythmias were associated with equal frequency in both patients with internal and real PE, whereas in none of the HC. The length of the anterior mitral leaflet did not differ between patients with internal or real PE, but was shorter in HC (p = 0.01 vs real PE and p = 0.02 vs internal PE). MVP was found more frequently in patients with internal or real PE (internal PE 24 (29%) vs real PE 4 (16%), p = 0.25), but not in HC.

##### CMR-FT

There were no differences between patients with internal or real PE and HC regarding LV myocardial strain analysis. Only RV-GLS was found to be higher (which means abnormal) in both patients with internal and real PE compared to HC ((%) internal PE − 11 ± 10 vs HC − 15 ± 4, p = 0.03; real PE − 10 ± 12 vs HC − 15 ± 4, p = 0.02). In addition, RV-GRS was reduced in patients with real PE compared to HC ((%) 20 ± 5 vs HC 27 ± 14, p = 0.02).

Table 2 provides a complete overview on CMR characteristics of patients with an internal or real PE and HC.

In order to investigate the clinical syndrome of patients with an internal PE in more detail and to reveal possible interrelationships, a correlation analysis was performed. The correlation matrix is displayed in Table 3. The analysis revealed a negative correlation of RV dimensions with thoracic indices (r = − 0.41) and symptoms (r = − 0.40), reflecting more RV compression as a result from a more pronounced thoracic constriction. Greater RA dimensions were associated with a larger contact zone (r = 0.27) and a higher amount of extrasystoles (r = 0.35). Thoracic indices were positively correlated with the intracardiac angles (r = 0.61) and the contact zone (r = 0.23) as well as ECG axis deviation (r = 0.26) and symptoms (r = 0.45). ECG axis deviation resulted in extrasystoles (r = 0.21) and was found more frequently in patients with large intracardiac angles (r = 0.25). MVP was more prevalent in patients with greater thoracic indices (r = 0.24) and larger intracardiac angles (r = 0.29). Pericardial effusion was also associated with greater thoracic indices (r = 0.30) and was found more frequently in female patients.

**Table 3:**
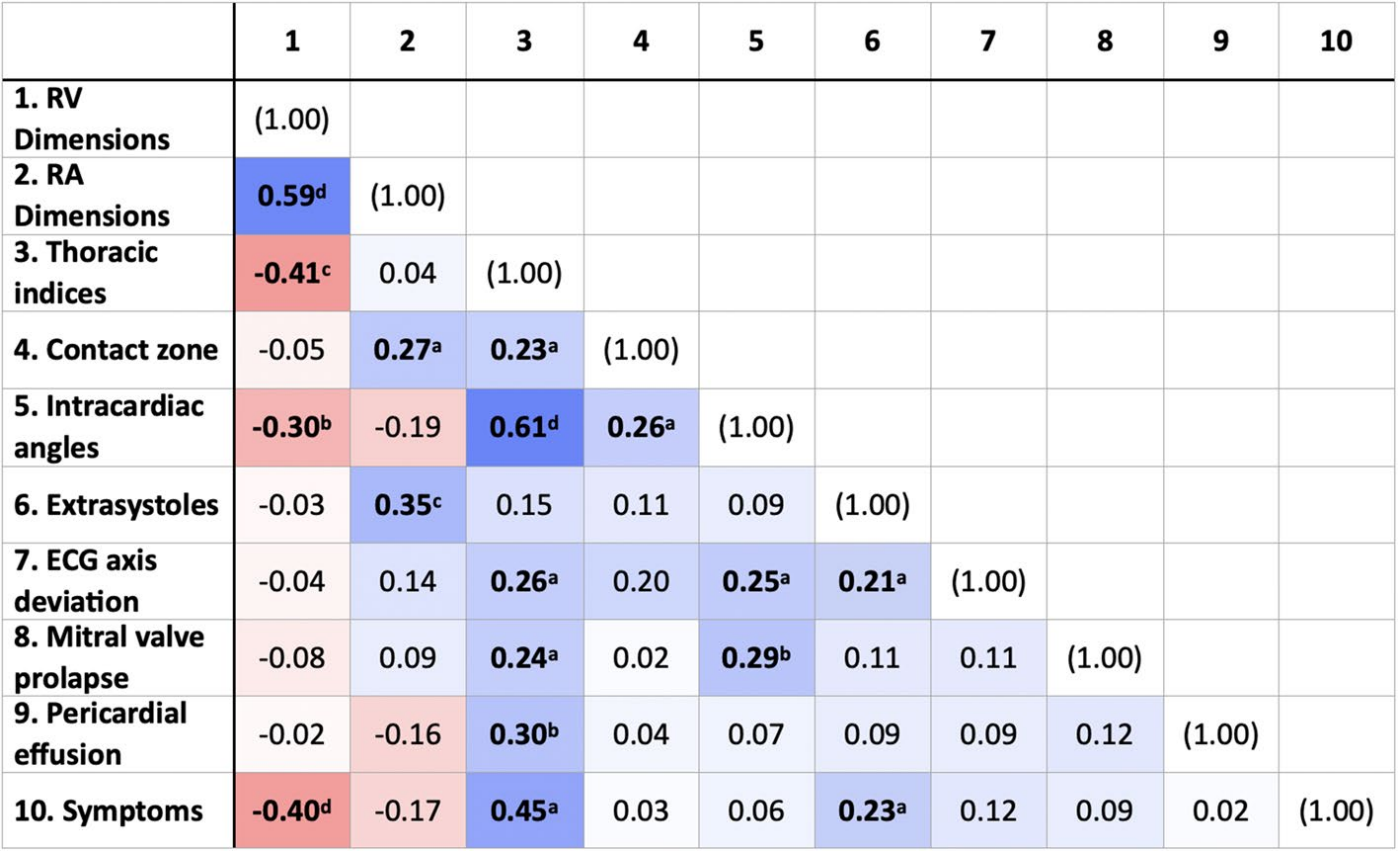
Correlation matrix.

Pooled within-group correlation matrix (internal PE, n = 88). Positive correlations are displayed in blue and negative correlations in red color, whereby color intensity is proportional to Pearson’s correlation coefficient r. Coefficients printed in bold are significant (^a^p < 0.05, ^b^p < 0.01, ^c^p < 0.001, ^d^p < 0.0001).

*ECG* electrocardiogram, *RA* right atrium, *RV* right ventricle.

### Follow-up of patients with internal PE

Clinical follow-up was available in 73 patients (83%). Mean follow-up time was 6.0 ± 4.1 years. Fifty-four patients (61%) complained of persistent symptoms. To treat these symptoms, ablation was performed in 12 patients, 10 patients received drug treatment (mainly based on betablockers), PE repair surgery was performed in another four patients and one patient was provided with a secondary prophylactic ICD. One patient died during follow-up, which was classified as death of cardiac origin, confirmed by autopsy.

## Discussion

This paper describes a case series of 88 patients evaluated by CMR for nonspecific cardiac complaints. The common feature in all patients was a “relative” thoracic constriction similar to PE, but without evidence of the pathognomonic funnel-shaped depression of the sternum, which is why the group was referred as “internal PE”. The following observations could be made, which will be further discussed below:

- The most common symptom were palpitations (41%) with documented SVES in 15% and VES in 21% of patients with internal PE.
- Atrial fibrillation or atrial flutter was present in 3%.
- Concomitant phenomena were pericardial effusion in 39% and MVP in 27% of the study cohort.
- Patients with an internal PE had a lower RVEF compared to HC (p < 0.01). Accordingly discrete changes in RV strain were found.
- RV dimensions were negatively correlated with the size of thoracic indices (r = − 0.41), reflecting the extent of thoracic constriction.
- MVP was more prevalent in patients with greater thoracic indices (r = 0.24).

### Pathogenesis

PE is the most common congenital chest wall deformity with an incidence of 1:300 to 1:400, while males are more often affected^1, 21^. The underlying embryological development is not clear. Abnormal growth of the sternocostal cartilages is discussed^22^. Although a genetic predisposition seems likely, as a positive family history can be found in up to 53%, a specific genetic defect has not yet been detected^23, 24^. Frequently, chest wall deformities occur as a single anomaly. However, associations with connective tissue disorders or other genetic defects have also been described^25^. The most important syndromes in which PE is found, are Marfan- or Noonan syndrome. PE can be seen in approximately two-thirds of patients with Marfan, whereas higher aortic root Z-scores were found in patients, presumed to have isolated PE, compared to HC^26^. In addition, MVP and mitral valve regurgitation has also been described in patients with real PE^27–29^. MVP was also a frequent finding in 27% of our cohort of patients with an internal PE. The association of idiopathic prolapse of the mitral valve with PE may represent a subtype of Marfan's syndrome according to Salomon et al.^29^. Another study examined the incidence of MVP in subjects with various thoracic skeletal abnormalities, including patients with narrow anteroposterior diameter of the chest (asthenic habitus), such as our cohort, straight back syndrome and real PE^30^. In this mixed cohort MVP was present in 31%. This high coincidence could be causally related to a growth pattern defect during embryonic development, since the primordia of the mitral valve undergo differentiation to their final form at the same time that the vertebral column and the thoracic cage are beginning their chondrification and ossification^31^. Furthermore, MVP is also known to be associated with connective tissue disorders, similar to PE, which makes a common genetic basis likely^32, 33^.

MVP is the most frequent valvular abnormality with a 2–3% prevalence in the general population^34^. Despite the general assumption that it is a harmless disease, some studies could demonstrate an increased risk of ventricular arrhythmias^35, 36^, which could also be detected in two of our patients.

### Cardiac arrhythmias

The present cohort had a mean Haller index of 3.9, formally meeting the criteria for a mild form of PE. In contrast, the mean Haller index of patients with a real PE was 6.6, which reflects the severity of the deformity. As CMR was performed in the majority of patients with a real PE as part of the routine work-up, prior to a corrective surgical repair, most of the patients were asymptomatic in contrast to patients with an internal PE, who underwent CMR for further evaluation of the heart, due to cardiac symptoms. The most common symptom were palpitations. Cardiac arrhythmias are also occasional findings in patients with real PE, which most likely results from a compression of the RV and a consecutive enlargement of the RA^3, 37^. This could also be observed in patients with internal PE. The arrhythmias associated with real PE are usually atrial fibrillation or supraventricular tachycardia^38, 39^. Three patients (3.4%) with internal PE had atrial fibrillation or atrial flutter, while the mean age of our study population was 35 years. An analysis of data of almost 8.3 million members of two German statutory health insurances revealed a significantly lower prevalence of 0.1% for atrial fibrillation in this age group^40^. In young patients, atrial fibrillation is frequently associated with congenital or structural heart diseases^41^. That PE is not just a cosmetic problem is demonstrated by the findings that PE can mimic other diseases. In approximately one third of patients with a real PE, abnormalities of the right heart, that have also been described in arrhythmogenic RV-CMP, can be found^13, 42^, as well as ECG changes (such as Brugada-like pattern)^43^. Even though primarily harmless supraventricular arrhythmias have been described in the context of PE, there are also some case reports of ventricular arrhythmias^44–46^. Two patients of our cohort (2%) also had a positive history of ventricular arrhythmias, which is a serious condition with potentially lethal consequences. So far, no clear explanation for the development of ventricular arrhythmia in patients with PE exists. Presumably, the mechanical compression of the RV and the cardiac rotation and anatomical displacement lead to conduction disorders^43^ or alterations of the epicardial tissue that act as a substrate for the arrhythmia^44^. This hypothesis could be supported by the fact that in some cases the arrhythmia could be resolved by surgical correction of the PE^44, 45^. Another explanation could be a potential common underlying genetic abnormality^39^. However, this has not been proven yet. Alternatively, the arrhythmia could be due to a MVP, as described above. This would also be supported by the high rate of LGE in our cohort of patients with internal PE, which occurs in approximately 50% of MVP patients and is often associated with complex arrhythmias^47, 48^.

### Cardiac irritation

Pericardial effusion was observed in 39% of patients with an internal PE. A high prevalence of pericardial effusion as echocardiographic finding in patients with real PE has also been described before^13, 49^. Possible explanations for this could be the lack of or insufficient fluid resorption caused by compression or increased fluid production due to irritant effects by contact with the osseous/ cartilaginous structures (contact zone). Usually, these pericardial effusions are not hemodynamically relevant and do not require surgical intervention. This was also shown in a recent CT study with a mean follow-up of 6.5 years^50^.

### Mechanical problem

Compared with HC, differences were found in RV function and dimensions, whereas LV function was mostly unaffected. This is explained by the anatomy. Close contact with the sternum and the cartilaginous structures are compressing the RV. In addition, the LV has a larger muscular layer which makes it less susceptible to compression. A negative correlation of the RV dimensions with the size of thoracic indices was found, reflecting the extent of the thoracic constriction. This could also be demonstrated by Zens et al.^51^. Of note, the severity of the RV dysfunction on the other hand bares no strict relationship to the Haller-Index^52^, so that even patients with little compression complain of severe symptoms, similar to our cohort. Since the right heart is more difficult to visualize in echocardiography anyway and patients with thoracic deformities often tend to have atypical cardiac axes, CMR has a special role in the evaluation of this clinical picture.

Besides these findings, less striking differences could be observed for biventricular strain. LV strain did not differ neither between patients with internal or real PE nor between patients with internal PE or HC. Only discrete changes could be revealed for RV-GLS which was higher (which means abnormal) in both patients with internal and real PE compared to HC^53^. These results indicate a purely mechanical problem in the absence of structural changes. In contrast, several small studies in patients with real PE found abnormal values for both LV and RV strain^14, 54, 55^, some of which were improved after surgical correction^56^. In addition, it is discussed whether the impairment of the strain is actually due to subclinical myocardial dysfunction or rather reflects intraventricular dyssynchrony due to the compression^57^. In the ECG, the dyssynchrony manifests as a bundle branch block, which was also present in four patients with internal PE.

### Management

More than 60% of patients reported persistent symptoms during the follow-up period, among these most often palpitations. Therefore, Holter recordings were performed to objectify the burden. Both interventional approaches (ablation of supraventricular or ventricular arrhythmias) and drug therapy were used to treat symptoms. One patient with documented VF and survived cardiac arrest was provided with a secondary prophylactic ICD. In four patients who reported disabling symptoms, PE repair surgery was performed as an individual healing attempt, with promising results. To what extent PE repair surgery should be used as a general treatment strategy cannot be answered on the basis of the available data. Further clinical studies are required to investigate this issue.

## Conclusions

The described cohort, referred to as internal PE because of the absence of external changes, showed similar CMR morphologic findings as patients with real PE. Above all, altered dimensions of the right heart presumably due to the compression could be detected, resulting in a lower RVEF, within the normal range. However, this seems to be a primarily mechanical problem in the absence of structural changes, since only discrete changes in biventricular myocardial strain analysis could be revealed. Most patients complained symptoms such as palpitations, dyspnea or atypical chest pain and in addition to a high incidence of rhythm disturbances, such as extrasystoles or supraventricular arrhythmias. Two patients even exhibited life-threatening VF and one patient died during follow-up, presumed to be of cardiac origin. In one-third of the study cohort additional abnormalities such as pericardial effusion or MVP were present, with MVP being found more frequently in patients with larger thoracic indices, suggesting a possible common pathogenesis.

Although the majority of our cohort reported cardiac symptoms, echocardiography was unremarkable in most cases. A main advantage of CMR, in addition to better visualization of the right heart, is the ability to assess the anatomical relationship and thoracic dimensions, in contrast to echocardiography. In view of the rather younger age of the presented cohort, it is important to take cardiac symptoms seriously and to consider a CMR in the case of an unremarkable echocardiography, as this may provide further information. Ablation strategies and drug treatment can help reduce symptoms and increase physical performance. In some patients with markedly severe symptoms, PE repair surgery yielded promising results. Whether PE repair surgery should be performed as a standard procedure, and possibly could even prevent life-threatening events, requires further investigation.

## Methods

This study was performed at the First Medical Department, University Medical Centre Mannheim, University of Heidelberg, Germany. Patients were included from January 2004 until June 2020 and followed prospectively.

The study was conducted according to the principles of the declaration of Helsinki and was approved by the local ethical committee, Medical Ethic Commission II, Faculty of Medicine Mannheim, University of Heidelberg, Germany (2020-800R). Data protection was in accordance with the EU Data Protection Directive.

### Study population

88 individuals who underwent CMR for further evaluation of the heart due to cardiac symptoms (such as palpitations, dyspnea, atypical chest pain, syncope) were enrolled. 25 patients with confirmed PE and 25 healthy individuals matched for age and sex served as controls (healthy controls, HC). All volunteers showed no contraindications for CMR and fulfilled the following criteria: normal physical examination, normal blood pressure (< 120 and < 80 mmHg), normal ECG findings, no history of chest pain or dyspnea, no diabetes, and normal two-dimensional echocardiography. None of the HC was on medication. Exclusion criteria were the symptoms of cardiac diseases, hypertension, diabetes, smoking, or participation in competitive or excessive sports. All control subjects underwent CMR examination using the same protocol.

Information on gender, age, height and weight (to calculate the body surface area (BSA)) were obtained. In addition, electrocardiogram (ECG) and in some cases Holter recordings were performed to detect rhythm events. Cardiac symptoms were evaluated in detail using a questionnaire.

### CMR image acquisition

All studies were performed using a 1.5 T whole body imaging system (Magnetom Avanto and Sonata, Siemens Medical Systems, Healthcare Sector, Erlangen, Germany) using a four-element (Sonata) or a six-element (Avanto), phased-array body coil. Images were acquired during repeated end-expiratory breath-holds. Scout images (coronal, sagittal, and axial planes) were obtained for planning of the final double-oblique long-axis and short-axis views. To evaluate functional parameters, ECG-gated cine images were then acquired using a segmented true fast imaging steady-state free-precession (TrueFISP) sequence [echo time (TE)/repetition time (TR) 1.2/3.2 ms, temporal resolution 35 ms, in-plane spatial resolution 1.4 × 1.8 mm^2^, slice thickness 6 mm, interslice gap 4 mm]. Seven to 12 short-axis views covering the whole left ventricle (LV) and RV were obtained. A T2-weighted half-Fourier acquisition single shot turbo spin echo (HASTE) sequence was acquired to obtain anatomically correct images of the thorax, from which the thoracic indices and intracardiac angles were derived^14^.

### CMR image analysis

CMR images were analyzed offline by two experienced physicians (AH and TP), blinded to all clinical patient details. Cardiac functional and morphological analysis was performed using dedicated commercially available software (Circle Cardiovascular Imaging Inc., CVI42, Calgary, Canada). Cardiac functional analysis included assessment of LV and RV ejection fraction (EF), LV/RV end-diastolic volumes (EDV), LV/RV end-systolic volumes (ESV), LV/RV stroke volume (SV) and 2D measurements of RV and right atrial (RA) dimensions, which were then compared to reference values (RV end-diastolic and end-systolic diameters, EDD/ESD)^15–17^. Late gadolinium enhancement (LGE) images were obtained 10–15 min after intravenous administration of 0.2 mmol/kg bodyweight Gadoteric acid (Dotarem, Guerbet, Germany), using T1-weighted inversion recovery gradient-echo sequences at the same position as the long and short-axis cine acquisitions in end-diastole^18^. Biventricular myocardial strain analysis by FT is a post processing method, that can be applied to routinely acquired cine CMR images^19^. Endocardial and epicardial borders on two- and four-chamber views, as well as short-axis for both LV and RV were drawn manually in end-diastole (reference phase) and two references points (superior and inferior septal insertion of the RV) were manually defined to allow accurate segmentation. The RV outflow tract wall in basal short axis slices was excluded from the strain calculations. Furthermore, the septum was not included in the evaluation of the RV strain. Strain measurements were automatically derived from a FT algorithm, which propagates the initial end-diastolic contours throughout the cardiac cycle^20^. Subsequently, global strain values are calculated for both LV and RV in different directions (circumferential (GCS), radial (GRS), longitudinal (GLS)).

### Thoracic indices

**Figure 2:**
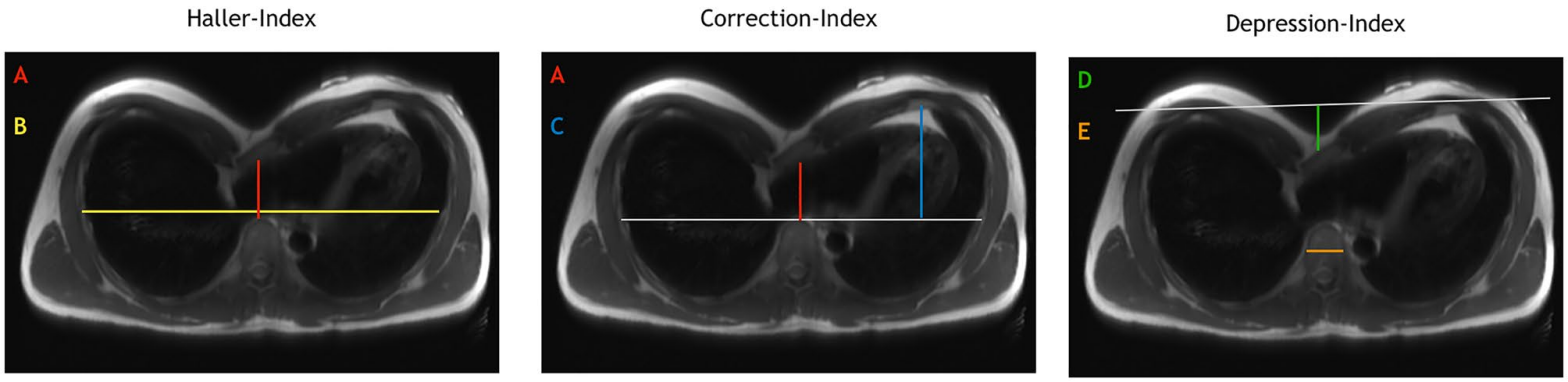
Thoracic indices. Schematic illustration of how to evaluate the different thoracic indices. Haller-Index (B/A): the thoracic diameter (B) divided by the distance from the sternum to the spine (**A**). Correction index ((C − A)/C*100): the difference of the largest inner dimension of the thoracic aperture in relation to a horizontal auxiliary line (grey line) (**C**) and the distance from the sternum to the spine (**A**) are put in relation to the inner dimension (**C**). Depression-Index (**D**/**E**): the depth of the sternal constriction (**D**) divided by the transverse diameter of the vertebral body.

There are different metrics to quantify a PE. The most common used is the Haller-Index^7^, indicating the ratio of the chest width to the deepest point of the sternal depression. As an evolution from the Haller-Index the Correction-Index attempts to reflect more precisely the severity of the PE by removing the chest width from the calculation^9^. The Correction-Index requires drawing a horizontal auxiliary line above the anterior spine. Then two distances are measured: the distance from the sternum to the vertebra and the greatest inner dimension of the thoracic aperture, from the costal arch to the horizontal auxiliary line. The difference between these two distances is put in relation to the inner dimension. The Depression-Index is based on the vertebral diameter, as morphometric correlate to the patient’s size^10^. To determine the Depression-Index, a line is drawn across the anterior ribs, from which a perpendicular is dropped to the point of maximum depression of the sternum. The depth of the sternal constriction is divided by the transverse diameter of the vertebral body at the corresponding level (Fig. 2).

### Intracardiac angles

**Figure 3:**
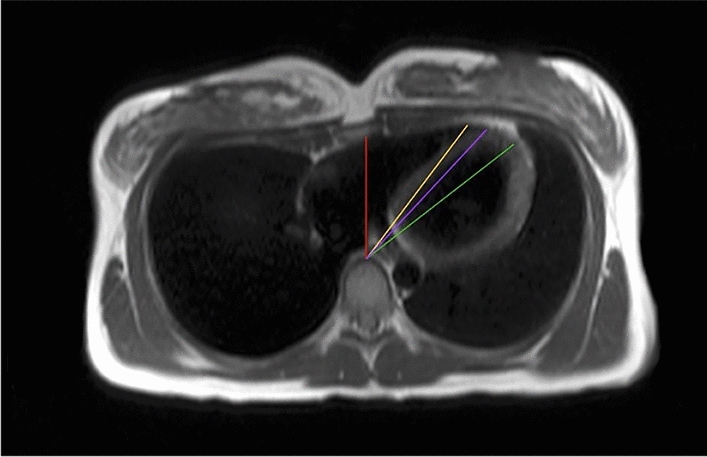
Intracardiac angles. Deviation of the heart axis was determined by measuring three different intracardiac angles, including the angle between the perpendicular of the thorax (red line) and the apex of the RV (yellow line)/insertion of the two ventricles (violet line)/apex of the LV (green line).

In addition to the thoracic indices, the contact zone of the heart with the osseous/cartilaginous parts of the thorax was measured, and different intracardiac angles (including the angle between the perpendicular of the thorax and the apex of the RV/insertion of the two ventricles/apex of the LV) were determined to quantify the deviation of the heart axis (Fig. 3).

### Follow-up of patients with internal PE

Follow-up was performed by telephone contact and regular visits in our cardiology outpatient department. Patients were screened for the occurrence of follow-up events (cardiac symptoms, arrhythmia, death). In addition, information on symptom management (drug treatment, ablation, PE repair surgery) was collected.

### Statistical analysis

Quantitative variables were evaluated using statistical parameters (n, arithmetic mean, standard deviation, median, minimum and maximum) and qualitative variables were evaluated in the form of frequency tables. Categorical variables were evaluated with the help of contingency tables. Comparisons between two groups were performed using a two-tailed Student's t-test for parametric and Mann–Whitney U test for non-parametric variables. Categorical variables were compared with the χ2 test or the Fisher exact test if the requirements of the χ^2^ test were not met. ANOVA was performed for comparison of averages between three groups and statistical differences were determined by Scheffé post hoc test.

All results were considered statistically significant when p < 0.05. Analyses were performed with Statistical Package for Social Sciences (SPSS for Windows 22.0, Chicago, IL, USA) and GraphPad Prism 9.0 (Graphpad Software, Inc., California, USA). All analyses are of explorative nature.

## Data Availability

Datasets are available from the corresponding author upon reasonable request.

## Abbreviations

BSA: Body surface area
CMP: Cardiomyopathy
CMR: Cardiac magnetic resonance imaging
ECG: Electrocardiogram
EDD: End-diastolic diameter
EDV: End-diastolic volume
EF: Ejection fraction
ESD: End-systolic diameter
ESV: End-systolic volume
FT: Feature tracking
GCS: Global circumferential strain
GLS: Global longitudinal strain
GRS: Global radial strain
HASTE: Half-Fourier acquisition single shot turbo spin echo
LGE: Late gadolinium enhancement
LV: Left ventricle
MVP: Mitral valve prolapse
PE: Pectus excavatum
RA: Right atrium
RAD: Right atrial diameter
RV: Right ventricle
SV: Stroke volume
SVES: Supraventricular extrasystole
TAPSE: Tricuspid annular plane systolic excursion
TE: Echo time
TR: Repetition time
TrueFISP: True fast imaging steady-state free-precession
VES: Ventricular extrasystole
VF: Ventricular fibrillation
